# Retraction: Heteroatoms (Si, B, N, and P) doped 2D monolayer MoS_2_ for NH_3_ gas detection

**DOI:** 10.1039/d5ra90040a

**Published:** 2025-04-04

**Authors:** Terkumbur E. Gber, Hitler Louis, Aniekan E. Owen, Benjamin E. Etinwa, Innocent Benjamin, Fredrick C. Asogwa, Muyiwa M. Orosun, Ededet A. Eno

**Affiliations:** a Computational and Bio-Simulation Research Group, University of Calabar Calabar Nigeria louismuzong@gmail.com; b Department of Pure and Applied Chemistry, Faculty of Physical Sciences, University of Calabar Calabar Nigeria; c Department of Chemistry, Akwa-Ibom State University Uyo Nigeria; d Department of Physics, University of Ilorin Ilorin Nigeria

## Abstract

Retraction of ‘Heteroatoms (Si, B, N, and P) doped 2D monolayer MoS_2_ for NH_3_ gas detection’ by Terkumbur E. Gber *et al.*, *RSC Adv.*, 2022, **12**, 25992–26010, https://doi.org/10.1039/D2RA04028J.

The Royal Society of Chemistry hereby wholly retracts this *RSC Advances* article due to evidence of systematic manipulation of the publication process. A number of references were inappropriately replaced with self-citations by the authors during the revision process.

In addition, ref. 31 and 35–39, the first reference in 48 and ref. 51 are irrelevant and/or inappropriate.

Given the significance of these concerns, the Editor has lost confidence in the authenticity of the findings presented in this paper.

The authors were informed but have not responded to any correspondence regarding the retraction.

Signed: Laura Fisher, Executive Editor, *RSC Advances*

Date: 28th March 2025

